# Role of Bunyamwera Orthobunyavirus NSs Protein in Infection of Mosquito Cells

**DOI:** 10.1371/journal.pntd.0001823

**Published:** 2012-09-27

**Authors:** Agnieszka M. Szemiel, Anna-Bella Failloux, Richard M. Elliott

**Affiliations:** 1 Biomedical Sciences Research Complex, School of Biology, University of St. Andrews, North Haugh, St. Andrews, Scotland, United Kingdom; 2 Department of Virology, Institut Pasteur, Paris, France; Centers for Disease Control and Prevention, United States of America

## Abstract

**Background:**

Bunyamwera orthobunyavirus is both the prototype and study model of the *Bunyaviridae* family. The viral NSs protein seems to contribute to the different outcomes of infection in mammalian and mosquito cell lines. However, only limited information is available on the growth of Bunyamwera virus in cultured mosquito cells other than the *Aedes albopictus* C6/36 line.

**Methodology and Principal Findings:**

To determine potential functions of the NSs protein in mosquito cells, replication of wild-type virus and a recombinant NSs deletion mutant was compared in *Ae. albopictus* C6/36, C7-10 and U4.4 cells, and in *Ae. aegypti* Ae cells by monitoring N protein production and virus yields at various times post infection. Both viruses established persistent infections, with the exception of NSs deletion mutant in U4.4 cells. The NSs protein was nonessential for growth in C6/36 and C7-10 cells, but was important for productive replication in U4.4 and Ae cells. Fluorescence microscopy studies using recombinant viruses expressing green fluorescent protein allowed observation of three stages of infection, early, acute and late, during which infected cells underwent morphological changes. In the absence of NSs, these changes were less pronounced. An RNAi response efficiently reduced virus replication in U4.4 cells transfected with virus specific dsRNA, but not in C6/36 or C7/10 cells. Lastly, *Ae. aegypti* mosquitoes were exposed to blood-meal containing either wild-type or NSs deletion virus, and at various times post-feeding, infection and disseminated infection rates were measured. Compared to wild-type virus, infection rates by the mutant virus were lower and more variable. If the NSs deletion virus was able to establish infection, it was detected in salivary glands at 6 days post-infection, 3 days later than wild-type virus.

**Conclusions/Significance:**

Bunyamwera virus NSs is required for efficient replication in certain mosquito cell lines and in *Ae. aegypti* mosquitoes.

## Author Summary

Bunyamwera and serologically related viruses are widely distributed in tropical and sub-tropical regions, and cause febrile illness in man. The viruses possess a trisegmented genome and can evolve by genetic reassortment generating viruses with different pathogenicity, like Ngari virus, a reassortant between Bunyamwera and Batai viruses, which causes haemorrhagic fever in humans. Like other arthropod-transmitted viruses, Bunyamwera virus can replicate efficiently in both mosquito and mammalian cells. Infected mammalian cells are killed by the virus whereas mosquito cells become persistently infected. Understanding the molecular basis for this difference may be crucial in designing new approaches to control bunyavirus disease. The viral non-structural NSs protein is the major virulence factor, which counteracts the innate immune defences of mammalian cells. In contrast, the role of this protein during infection of vector mosquito cells is unknown. We compared the replication of wild type virus and a genetically engineered virus that does not express NSs in various cultured mosquito cell lines and in *Aedes aegypti* mosquitoes. We showed that some cells did not support mutant virus replication, implying a role for the NSs protein. NSs protein was also important for efficient replication and dissemination in potential vector species.

## Introduction

Bunyamwera virus (BUNV) is the prototype of both the *Orthobunyavirus* genus and the *Bunyaviridae* family. It was originally isolated from a pool of several *Aedes* spp. mosquitoes collected in the Semliki Forest in Uganda [1]. Based on detection of antibodies to BUNV in human sera and isolations of BUNV from patients suffering febrile illness, the virus is widely distributed in several regions of sub-Saharan Africa [2]–[4]. BUNV is maintained in nature by a propagative cycle involving blood-feeding mosquitoes and susceptible vertebrate hosts, probably small rodents [5]. BUNV can replicate efficiently in both vertebrate and invertebrate cells in culture but with different outcomes: in mosquito cells no cytopathology is observed and persistent infection is established, whereas in mammalian cells infection is lytic and leads to cell death [6]–[8]. From a practical standpoint, this is shown by the ability of the virus to form clear lytic plaques in cells of vertebrate origin but not in those derived from insects.

Like all bunyaviruses, BUNV is an enveloped virus containing a tri-segmented, single stranded negative-sense RNA genome that encodes four common structural proteins: an RNA-dependent RNA polymerase (L protein) on the large (L) segment, two glycoproteins (Gc and Gn) on the medium (M) segment and the nucleoprotein (N) on the smallest (S) segment. BUNV also codes for two non-structural proteins, NSm on the M segment and NSs on the S segment [9]. The segmented nature of the genome allows for reassortment between closely related orthobunyaviruses to generate viruses that may have altered biological properties, such as Ngari virus, which is associated with human haemorrhagic fever in East Africa, whose genome comprises L and S segments from BUNV and M segment from Batai virus [10]–[12]. BUNV continues to serve as a convenient laboratory model to study the molecular biology of bunyaviruses in general, and an understanding of many aspects of gene function and viral replication have been developed using BUNV, including the establishment of a robust reverse genetics system [13]–[15].

The BUNV NSs protein is a nonessential gene that contributes to viral pathogenesis. It has been shown that in mammalian cells, NSs induces shut-off of host protein synthesis [16], [17], which leads to cell death. It also counteracts the host cell antiviral response and seems to be the main virulence factor [18]–[21], acting at the level of transcription by inhibiting RNA polymerase II–mediated transcription [22]. In mosquito cells neither host cell transcription nor translation are inhibited [17], and although so far no function for the orthobunyavirus NSs protein has been found in mosquito cells [23], it seems the differential behaviour of NSs could be one of the factors responsible for different outcomes of infection in mammalian and mosquito cell lines.

To date, studies on BUNV replication in mosquito cells have been limited to the C6/36 line [24] from *Aedes albopictus* [6]–[8], [16], [17], [25]–[27]. As differences in the appearance of cytopathic effects, cell death and viral morphogenesis were observed in various *Ae. albopictus* subclones infected with Sindbis virus [28]–[32], we have compared the replication of BUNV in two additional *Ae. albopictus* cell clones, C7-10 [33] and U4.4 [34]. All three cell lines were independently obtained from the original Singh cell line derived from *Ae. albopictus* neonate larvae [35], which has been shown to comprise a heterogeneous population of cell types. More recently it has been reported that these cell lines differ in their RNAi responses: C7-10 [36] and C6/36 [37]–[39] cells have impaired Dicer 2-based RNAi responses, whereas the U4.4 cell line encodes a fully functional Dicer 2 gene [36], [40]. Secondly, we have investigated the ability of BUNV to replicate in *Aedes aegypti* (Ae) cells, and have compared this to the infection in living *Aedes aegypti* mosquitoes. Here we report that *Ae. albopictus* U4.4 and *Ae. aegypti* Ae cells are refractory to infection with a recombinant BUNV lacking the NSs gene (rBUNdelNSs2; [16], [17]), and that expression of NSs influences the efficiency of viral replication in *Ae. aegypti* mosquitoes.

## Materials and Methods

### Cells and viruses

*Aedes albopictus* C6/36, C7-10 and U4.4 and *Aedes aegypti* Ae cells were grown at 28°C in Leibovitz 15 medium (Gibco) supplemented with 10% foetal calf serum (FBS) and 10% tryptose phosphate broth. Vero E6 cells were maintained at 37°C in Dulbecco's modified Eagle's medium (Gibco) supplemented with 10% FBS. Working stocks of wild-type Bunyamwera virus (wtBUNV), the recombinant NSs deletion virus (rBUNdelNSs2; [17]), rBUNGcGFP [41] and rBUNdelNSs-GcGFP [42] were prepared as described previously [18], [43].

### Virus growth curves and titration

Mosquito cells were infected at an MOI of 1 PFU/cell. After 1 h incubation at 28°C, the inoculum was removed and the cells were washed once to remove unattached virus. Supernatants were harvested at various times post-infection and assayed for virus by plaque assay on Vero E6 cells as previously described [13], [44]. In our laboratory, we routinely titrate BUNV on Vero E6 cells as these give the most easily discernible plaques. We have also performed immunostaining assays of viral foci produced in C6/36 cells, and observed that the efficiency of plating compared to Vero E6 cells is in the range 0.5 to 1 (data not shown). This is similar to the plating efficiency for other arboviruses, like Dengue, West Nile and St Louis encephalitis viruses [45], when comparing titration on C6/36 and vertebrate cells.

### Analysis of protein by Western blotting

At different times after infection, cell lysates were prepared and equal amounts of sample were separated on 12% SDS-PAGE. Separated proteins were transferred to Hybond-C Extra membranes (Amersham). The membranes were incubated with rabbit anti-BUN N protein antibody (1∶2000 dilution) and mouse anti-tubulin antibody (Sigma; 1∶10000) as a loading control, followed by incubation with anti-rabbit horseradish peroxidase-coupled antibody (Cell Signaling Technology). Immunocomplexes on the membranes were detected by SuperSignal West Pico Substrate (Pierce) according to manufacturer's instructions.

### Indirect immunofluorescent staining

Mosquito cells were cultured on glass coverslips and infected with rBUNGceGFP or rBUNdelNSs-GcGFP at MOI of 1 PFU/cell. At various times post infection, cells were fixed with 4% formaldehyde and washed with PBS. The cells were incubated with rabbit anti-BUN N (1∶200) and mouse anti-tubulin (Calbiochem; 1∶100) antibodies, followed by incubation with Texas Red-conjugated anti-rabbit (Cell Signaling Technologies; 1∶200) and CY5-conjugated anti-mouse (Sigma; 1∶400) antibodies. Nuclei were stained with DAPI. Prepared slides were examined with a Zeiss LSM confocal microscope.

### Mosquito infections

Laboratory-bred *Ae. aegypti* (Paea strain) mosquitoes were reared as previously described [46]. Female mosquitoes were selected and exposed to blood-meal containing approx. 10^8^ PFU/ml of wtBUNV or rBUNdelNSs2 as described previously. At various times post infection, mosquitoes were anesthetized and either homogenized whole or the midguts and salivary glands were dissected. Organs and whole mosquitoes were homogenized in 100 µl of Leibowitz 15 medium (Gibco). Twenty-five microliters of each sample were used for titration by plaque assay on Vero E6 cells.

### Production of double-stranded RNA and transfection of mosquito cells

dsRNA approx. 1000 bp long was prepared using the Megascript RNAi kit (Ambion). Virus specific dsRNA were prepared from linearized plasmids containing full-length cDNAs of the BUNV genome segments pT7riboBUNS(+) and (−), pT7riboBUNM(+) and (−) and pT7riboBUNL (+) and (−) [13]. *Renilla*-specific dsRNA were prepared from plasmid phRL-CMV by adding a T7 promoter sequence at each end by PCR as described previously [40]. Purified dsRNA was stored in aliquots at −80°C. A total of 3×10^5^ cells per well were cultured in 24 well plates. 100 ng of *Renilla* dsRNA or 100 ng of a 1∶1∶1 mixture of S-, M-, and L-segment specific dsRNA was used for transfection. One microliter of Lipofectamine-2000 (Invitrogen) was used per 100 ng and the transfection mixes were prepared in the final volume of 100 µl as per the manufacturer's protocol; this was then applied onto the cells with 400 µl fresh complete L15 medium. After 5 hours incubation at 28°C, 500 µl of fresh medium was added. The cells were infected with wtBUNV or rBUNdelNSs2 24 h post-transfection, supernatants were collected at various times thereafter for assay of released virus by plaque titration on VeroE6 cells.

## Results

### Growth of BUNV in various *Ae. albopictus* cell lines

**Figure 1:**
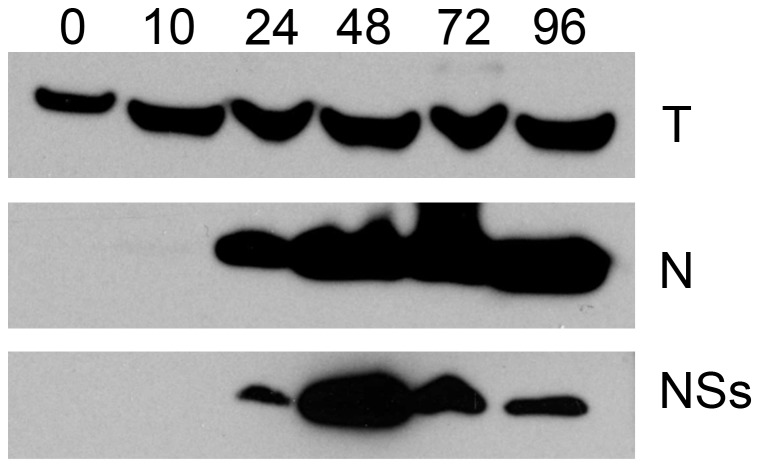
Expression of NSs protein in wtBUNV infected mosquito cells. *Ae. albopictus* C6/36 cells were infected at an MOI of 10 PFU/cell and samples were harvested at the indicated time points. Cell lysates were electrophoresed on a 4–12% NuPAGE gel (Invitrogen). After transfer the membrane was cut into three and strips were incubated with anti-BUNV NSs protein (NSs), anti-BUNV N protein (N) or anti-tubulin (T) antibodies as indicated.

In mosquito cells no shut-off of host cell transcription or translation has been observed. Therefore, we first confirmed that BUNV NSs protein is expressed in these cells. *Ae. albopictus* C6/36 cells were infected at MOI of 10 pfu/cell and the cells were harvested for protein analysis by Western blotting at different time points (Figure 1). Although BUNV N and NSs proteins are translated from the same mRNA, NSs was produced, though slightly later in the course of infection than the nucleoprotein. This is in line with previous observations [8] based on radiolabelling of infected cells.

**Figure 2:**
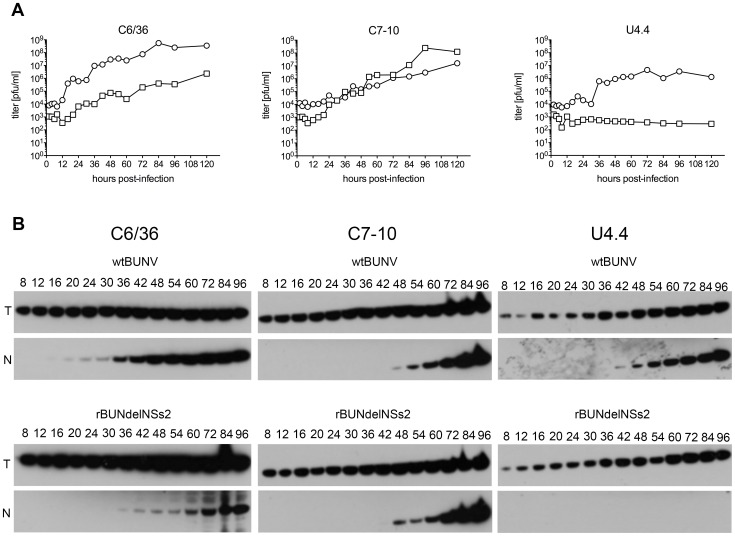
Growth of wtBUNV and rBUNdelNSs2 in *Aedes albopictus* cell lines. **A.** Growth curves. C6/36, C7-10 and U4.4 cells were infected at an MOI of 1 PFU/cell. At the indicated time points, supernatants were collected and viral titres were determined by plaque assay. **B.** Viral N protein expression. Cells were infected at an MOI of 1 and at the indicated time points (hpi), cells were lysed, and proteins separated by 12% SDS-PAGE. The proteins were transferred to a membrane and incubated with anti-BUNV N protein (N) or anti-tubulin (T) antibodies.

We next compared virus replication in *Ae. albopictus* C6/36, C7-10 and U4.4 cell lines. Cells were infected at an MOI of 1 PFU/cell and the samples were collected at various times post infection. wtBUNV was able to replicate in all three cell lines but with different kinetics (Figure 2A). Growth was most efficient in C6/36 cells, with maximum titres of released virus exceeding 10^8^ pfu/ml, 10-fold and 100-fold higher than in C7-10 or U4.4 cells, respectively. rBUNdelNSs2 grew more slowly than wtBUNV in C6/36 cells and yielded maximum titres about 100-fold less, confirming previous results [16], [17]. In C7-10 cells, the mutant virus showed similar kinetics to wt BUNV. In marked contrast, no increase in titre of rBUNdelNSs2 in culture medium of U4.4 cells was detected. (The detection of some rBUNdelNSs2 virus in supernatant medium (Figure 2A) represents residual virus that remained after removal of the inoculum and replacement with fresh medium; these cells did not adhere firmly enough to the culture vessel to permit extensive washing).

These data were supported by Western blot analysis to detect accumulation of the viral N protein (Figure 2B). In C6/36 cells infected with wtBUNV, N could be seen as early as 16 h post-infection (hpi), whereas in cells infected with the NSs deletion, mutant N was not detected until 36 hpi. In the C7-10 cell line, no significant difference between the two viruses was observed in terms of accumulation of N protein, though growth was slower than in C6/36 cells, with N protein first detectable at 48 hpi. In U4.4 cells infected with wtBUNV, N was detectable as early as 42 hpi whereas no N protein could be detected in cells infected with rBUNdelNSs2. These results suggest that BUNV NSs protein is not essential for growth in either C6/36 or C7-10 cell lines, but is required for successful replication in U4.4 cells.

### Morphological phases of infection in mosquito cells

**Figure 3:**
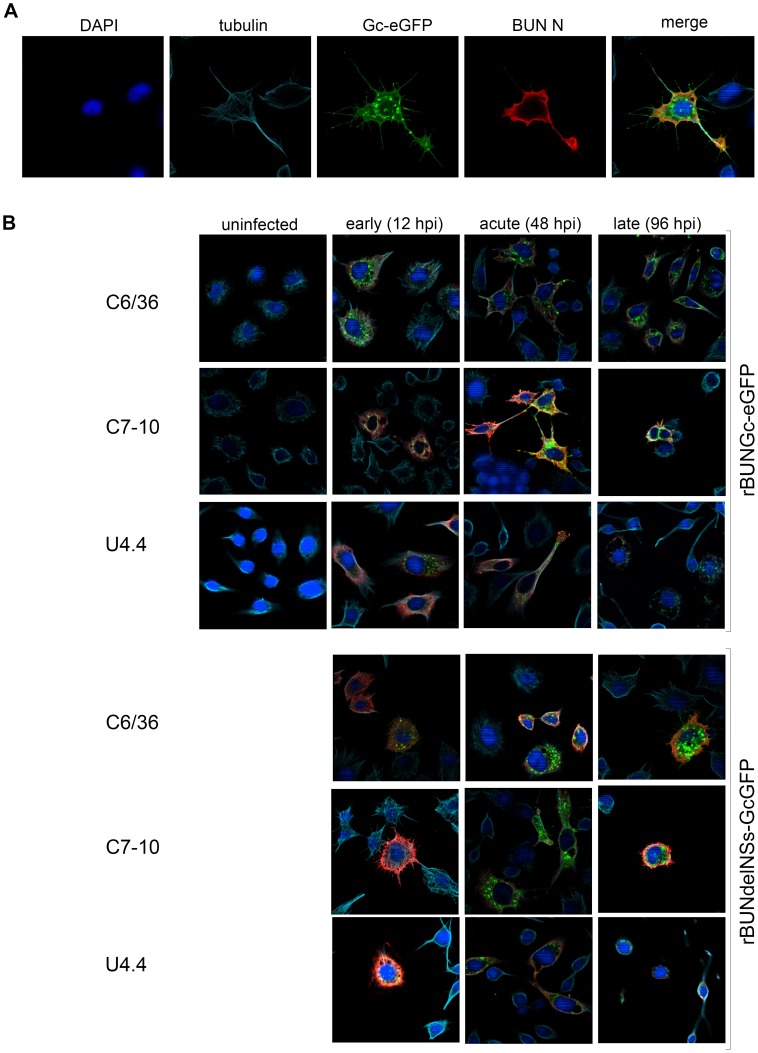
Immunofluorescence analysis of morphological changes in *Ae. albopictus* cell lines. Mosquito cells were infected with rBUN-GcGFP or rBUNdelNSs-GcGFP at an MOI of 3 PFU/cell. The results present cells stained with anti-BUNN/anti-rabbit TexasRed (red signal), and with anti-tubulin/anti-mouse CY5 (light blue signal). The green signal shows autofluorescence of GFP-tagged Gc and the blue signal is DAPI staining of nuclei. **A.** C6/36 cell infected with rBUN-GcGFP virus during the acute stage of infection. **B.** C6/36, C7-10 and U4.4 cells were infected with fluorescent viruses. Three morphological stages of infection are presented: early, acute, and late. Only the merged images are shown.

To analyse how BUNV spreads in mosquito cells, *Ae. albopictus* C6/36, C7-10 and U4.4 cells were infected with recombinant BUNV expressing green fluorescent protein [41], either rBUNGc-eGFP or rBUNdelNSs-GcGFP at an MOI of 3 PFU/cell. Three different stages of infection, as proposed by Lopez-Montero et al. (2009), were observed in all the *Ae. albopictus* cell lines infected with rBUNGc-eGFP. These phases were defined by changes in cell morphology due to virus replication, most notably that infected cells produced projections extending towards neighbouring cells (Figure 3A). During the early phase of infection with the wild type version of the eGFP-expressing BUNV, the levels of viral proteins were relatively low and the cells resembled uninfected cells in shape (Figure 3B). However, as infection progressed and the levels of viral proteins increased, cells transitioned into the acute phase. This phase was characterised by formation of filopodia that were most abundant between the 24 hpi and 48 hpi; over this time period, cells maintained physical contact via the projections. During the acute phase, the levels of eGFP-tagged Gc increased and the glycoprotein was also found in the filopodia (Figure 3B). The late phase of the infection was characterised when the cellular filopodia started to disappear (from 48 hpi), which was also manifested by elevated levels of Gc glycoprotein detected in the cells. Later on in the infectious cycle, the cells returned to their normal form (Figure 3B).

These results were consistent for infection of C6/36 and C7-10 cells. Infection in U4.4 cells was slower, and fewer cells were infected in comparison to C6/36 and C7-10 cells. Also, U4.4 cells produced fewer filopodia (Figure 3B). Comparison of the levels of viral proteins in infected cells revealed that BUNV replication in U4.4 cells was less intense than in C6/36 and C7-10 cell lines, as relatively lower amounts of N and Gc proteins were produced. These observations corresponded with the differences in viral titres observed in the previous experiments (Figure 2), where U4.4 cells produced less infectious particles than the two other cell lines.

The course of infection with the NSs deletion recombinant, rBUNdelNSs-GcGFP, was similar to rBUNGc-eGFP in terms of levels of viral protein expression, but the infection started later in C6/36 and C7-10 cells. Investigation of cell morphology showed that fewer filopodia were produced and they were less pronounced in rBUNdelNSs-GcGFP infected cells, suggesting involvement of NSs in this process. Minimal disruption of the normal cell morphology was observed; those changes that were seen were attributed to cell movement and consecutive attachment to the surface, as similar changes were observed in uninfected cells (Figure 3B). These data suggested that NSs protein contributed to the efficiency of viral replication, but was a non-essential protein.

When U4.4 cells were infected with rBUNdelNSs-GcGFP, a few cells (less than 5%) were observed to harbour replicating virus in that synthesis of tagged Gc protein could be observed by its autofluorescence (Figure 3B). This suggests that infection by rBUNdelNSs-GcGFP is abortive and few, if any, new infectious particles were produced, in line with the titration data shown in Figure 2.

In order to rule out the possibility that changes in morphology were due to alterations of Gc glycoprotein caused by fusion of the GFP sequences, another fluorescent Bunyamwera virus was used, with eGFP fused in-frame into NSm [46]. Infection of the C6/36, C7-10 and U4.4 cells with rBUNM-NSm-EGFP, but not with rBUNdelNSs-NSm-EGFP, resulted in similar marked morphological changes as shown by rBUNGc-eGFP (data not shown).

### BUNV establishes persistent infection in *Ae. albopictus* cell lines

**Figure 4:**
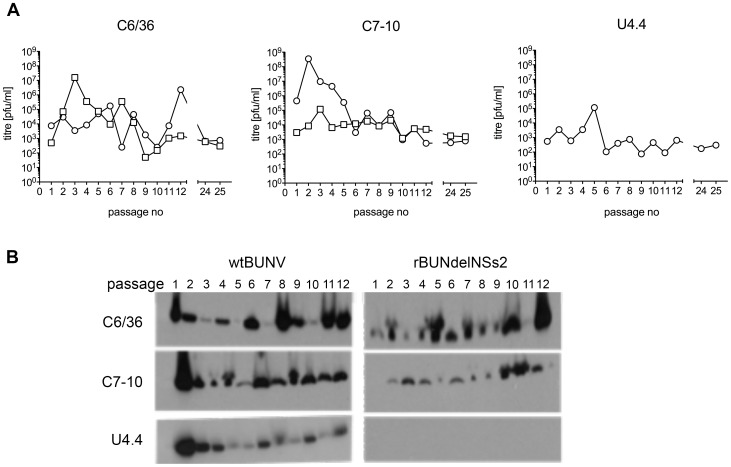
Establishment of persistent infection in *Ae. albopictus* cell lines. **A.** Titres of virus released into the supernatant from each passage of cells. Cells were initially infected at MOI of 0.1 PFU/cell, and passaged regularly at a 1∶5 split ratio. The supernatant medium was collected at the time of each passage and the presence of virus measured by plaque assay. **B.** Detection of viral N protein. An aliquot of cells was collected at each passage, lysed and 10 µg of total protein was analyzed by Western blotting with anti-BUNV N protein antibodies. Numbers at the top of the blots indicate the passage number.

It has been previously documented that the outcome of infection of C6/36 cells with wtBUNV is the establishment of a persistent infection [6]–[8]. To determine whether persistent infections could also be established in C7-10 and U4.4 cells, and if the NSs protein participates in this process, cell monolayers were initially infected with wtBUNV or rBUNdelNSs2 at an MOI of 0.1 PFU per cell. After four days, supernatant fluids were collected for titration of released virus, and the infected cells were passaged (split ratio of 1∶5) and grown until confluent. Thereafter, they were regularly passaged and maintained for about 7 months (25 passages). All lines initially infected with wtBUNV or rBUNdelNSs2 continued to shed infectious virus at each passage, though the titres fluctuated widely (Figure 4A). Similarly, N protein was detected by Western blotting in all passaged cells, though the levels varied. Thus wtBUNV could establish persistent infections in all three cell lines, and rBUNdelNSs2 in C6/36 and C7-10 cells. As expected, since U4.4 cells did not show evidence of productive infection by rBUNdelNSs2, no indication of a persistent infection was obtained. Thus the NSs protein was not essential for establishment of persistent infection in C6/36 or C7-10 cells.

In previous studies of C6/36 cells persistently infected with BUNV, the generation of viruses displaying different plaque phenotypes when titrated in mammalian cells was observed [6], [7]. Similarly, when titrating the two viruses released from all persistently infected cell lines described above in Vero cells, we observed the appearance of large, small and cloudy plaques from different passages (data not shown).

### Activation of Dicer 2 in U4.4 cells counteracts wtBUNV replication

**Figure 5:**
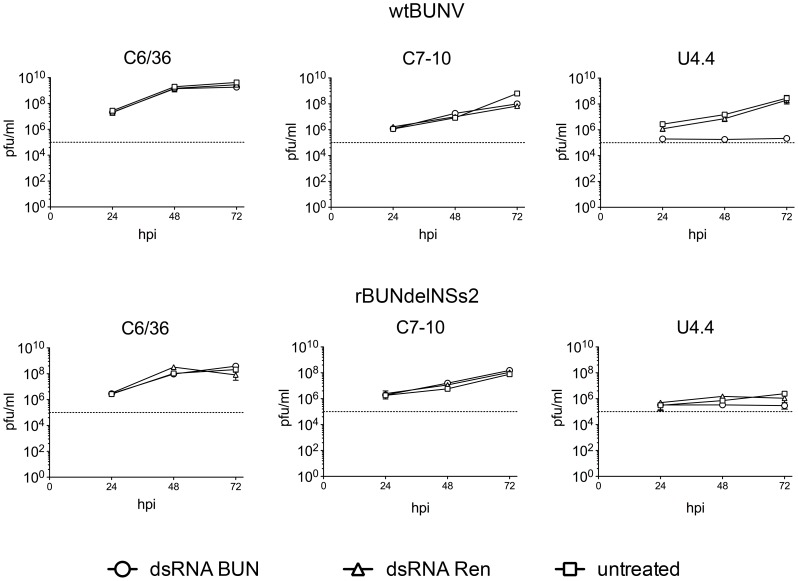
Dicer 2 activity in *Ae. albopictus* C6/36, C7-10 and U4.4 cells. Cells were transfected with BUNV-specific long dsRNA, *Renilla*-specific long dsRNA or left untransfected (untreated) as indicated. At 24 h post-transfection, cells were infected with wtBUNV or rBUNdelNSs2 at an MOI of 5 PFU/cell. Supernatants were collected at the indicated times post infection for determination of the presence of infectious virus by plaque assay.

As mentioned above C7-10 and C6/36 cells are reported to have defective Dicer 2-based RNAi responses [36]–[39], whereas the U4.4 cell line encodes a fully functional Dicer 2 gene [36], [40]. To determine whether the cells could mount an RNAi response against BUNV infection, we transfected C6/36, C7-10 and U4.4 cells with long virus-specific dsRNA, or *Renilla* luciferase-specific dsRNA as a control, and then infected the cells with either wtBUNV or rBUNdelNSs2 at a MOI of 5 PFU per cell. Supernatant fluids were collected at various times post-infection and assayed for the presence of infectious virus by plaque formation. Each infection was done in triplicate and the experiment was repeated twice. As shown in Figure 5, no effect on virus growth was seen in C6/36 or C7/10 cells infected with either virus. However, in U4.4 cells transfected with virus-specific dsRNA and then infected with wt BUNV, virus replication was inhibited, and no increase in virus titre was observed. In contrast, transfection of *Renilla* luciferase-specific dsRNA had no effect on wtBUNV growth in any cell line. These results are consistent with U4.4 cells having a functional dsRNA-mediated interference system. Unfortunately, because rBUNdelNSs2 virus does not replicate productively in U4.4 cells it was not possible to determine whether BUNV NSs protein could be involved in evasion of an RNAi response in mosquito cells.

### BUNV replicates and establishes persistent infection in the *Ae. aegypti* Ae cell line

**Figure 6:**
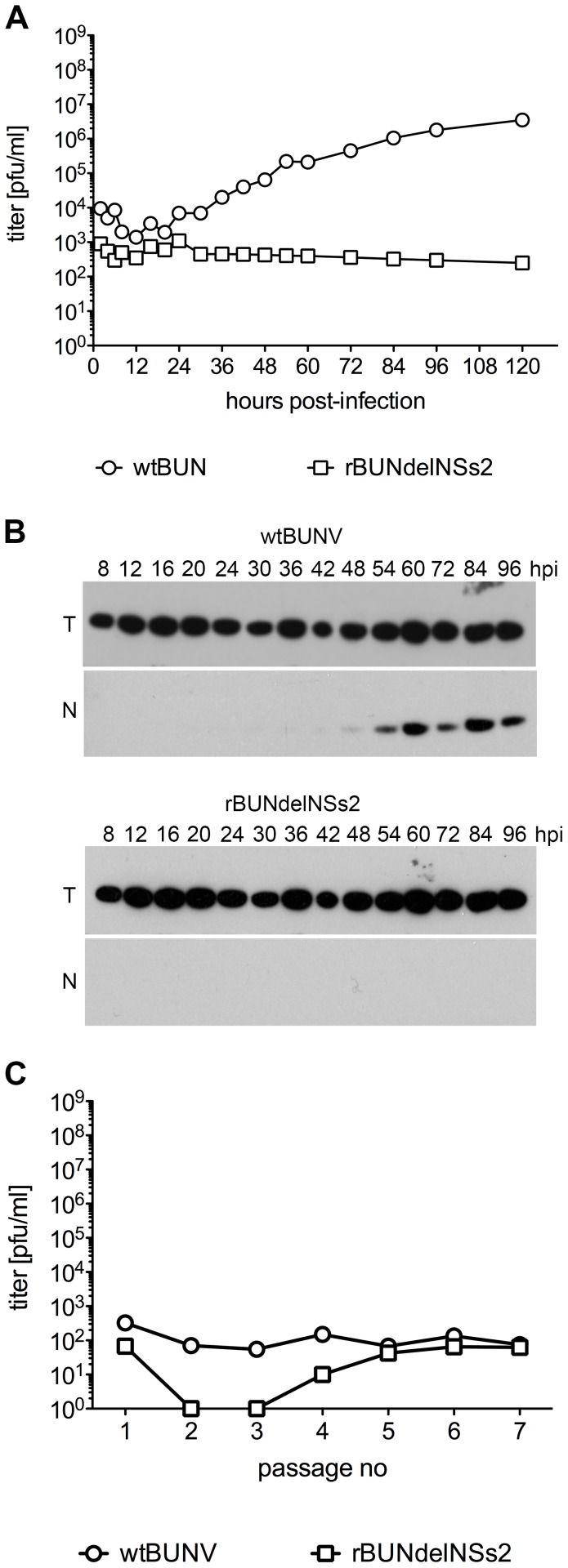
BUNV replication in *Ae aegypti* Ae cells. **A.** Growth curves. Ae cells were infected with wtBUNV or rBUNdelNSs2 at an MOI of 1 PFU/cell. At the indicated times post infection supernatants were collected and assayed for the presence of infectious virus by plaque titration. **B.** Viral N protein expression. Cell extracts were prepared at the indicated times (h P.I.), separated by 12% SDS-PAGE, and proteins transferred to a membrane. The membrane was incubated with anti-BUNV N protein and tubulin (T) antibodies. **C.** Establishment of persistent infection. The titres of infectious virus in supernatants collected at each passage of infected cells were determined by plaque assay.

Although *Ae. albopictus* cell lines are widely used to investigate arbovirus replication, the genome sequence of *Ae. albopictus* has yet to be determined, thus limiting detailed investigation of molecular details of virus-host interaction. To date three mosquito genome-sequencing projects have been completed, *Anopheles gambiae*, *Aedes aegypti* and *Culex quinquefasciatus* [47]–[49], and hence we examined whether cell lines from other mosquito species would be useful to monitor BUNV replication. In preliminary experiments, no evidence for BUNV growth in the Sua4 cell-line derived from *An. gambiae* Suakoko strain [50] was obtained (data not shown). However, the Ae cell line [51] derived from *Ae. aegypti* was shown capable of supporting wtBUNV replication (Figure 6). The virus grew to titres approaching 10^7^ PFU/ml, and accumulation of BUNV N protein from 48 hpi was detected by Western blotting. In contrast, rBUNdelNSs2 appeared unable to replicate in these cells, as N protein was not detected by Western blotting (Figure 6B) and no increase in titre of infectious virus in the supernatant medium was measured. (Again as these cells did not adhere firmly to the culture vessel, extensive washing to remove the virus inoculum was not possible, and only residual infecting virus was detected).

To investigate if BUNV was capable of establishing persistent infection in the Ae cell line, cells were infected with wtBUNV or rBUNdelNSs2 at an MOI of 0.1 PFU per cell. Supernatants were collected and infected cells were then passaged using a fifth of gathered cells as it was done with *Ae. albopictus* cell lines. Cells were maintained for 7 passages. Analysis for the presence of infectious virus in the supernatants showed the Ae cell line to be persistently infected with wtBUNV (Figure 6C). However the levels of infectious virus production remained low and there was no significant difference between consecutive passages as was seen in *Ae. albopictus* cell lines. Infection with rBUNdelNSs2 showed a different pattern. Infectious virus particles were detected after the first passage, but no virus was detected in the supernatants for the next two passages. However, from passage 4, low titres of virus were detected (Figure 6C). This pattern was reproducibly observed in two further independent infections of Ae cells with rBUNdelNSs2, and also when another *Ae. Aegypti* cell line, A20 [51] was infected with rBUNdelNSs2: in all cases no virus could be detected by plaque assay of supernatants from the second and third passages, but virus was detected at subsequent passages at low levels (data not shown).

### NSs is required for efficient replication and spread in mosquitoes

Having demonstrated that BUNV could replicate in *Ae. aegypti* cell cultures, we next studied the infection of *Ae. aegypti* mosquitoes. It has been reported previously that wtBUNV is capable of replicating in laboratory raised *Ae. aegypti* mosquitoes and of being transmitted via mosquito saliva to mice [52]. By comparing infection with wtBUNV and rBUNdelNSs2, we investigated the role of NSs in an insect vector. Female *Ae. aegypti* (Paea strain) mosquitoes were allowed to feed on a blood-meal containing wtBUNV or rBUNdelNSs2 (approx. 10^8^ PFU/ml in the blood meal). After feeding, only engorged mosquitoes were kept for the course of the experiment. Three engorged females were collected from each infection group immediately after feeding to determine the amount of virus ingested. Based on the titration results, it was estimated that each mosquito imbibed two to three microliters of blood (data not shown). These blood-meal volumes were similar to those previously published for various mosquito species, including *Ae. aegypti* [53]–[56]. The survival rates following the blood-meal were calculated by recording the number of dead mosquitoes daily. Survival rates for each virus were above 98%, which suggests that neither wtBUNV nor rBUNdelNSs2 had any detrimental effects on mosquito viability.

**Figure 7:**
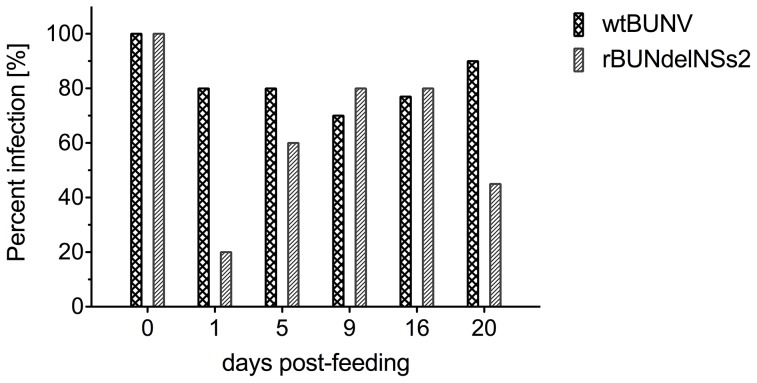
Infection of female *Ae. aegypti* mosquitoes with wtBUNV or rBUNdelNSs2. Following infection via blood-meal, 8 to 10 engorged mosquitoes were collected at the indicated days after feeding, individually homogenized and the presence of infectious virus determined by plaque assay. Mosquito infection rates were calculated as the percentage of virus positive females over total number of engorged mosquitoes tested.

At various times post feeding, 8 to 10 female mosquitoes were collected, homogenised and the levels of infectious virus they contained were titrated by plaque assay. Infection rates were calculated by dividing the number of infected female mosquitoes by the total number of engorged mosquitoes tested at a given day post-feeding (Figure 7). For wtBUNV, >70% of mosquitoes contained infectious virus at all time points. In contrast, at day 1 after feeding, only 20% of mosquitoes fed rBUNdelNSs2 showed evidence of infectious virus. However, by 5 days post-feeding, virus was detected in 60% of mosquitoes, and this rose to 80% by day 9 (Figure 7). Thus the lack of NSs seemed to delay the progress of infection.

**Figure 8:**
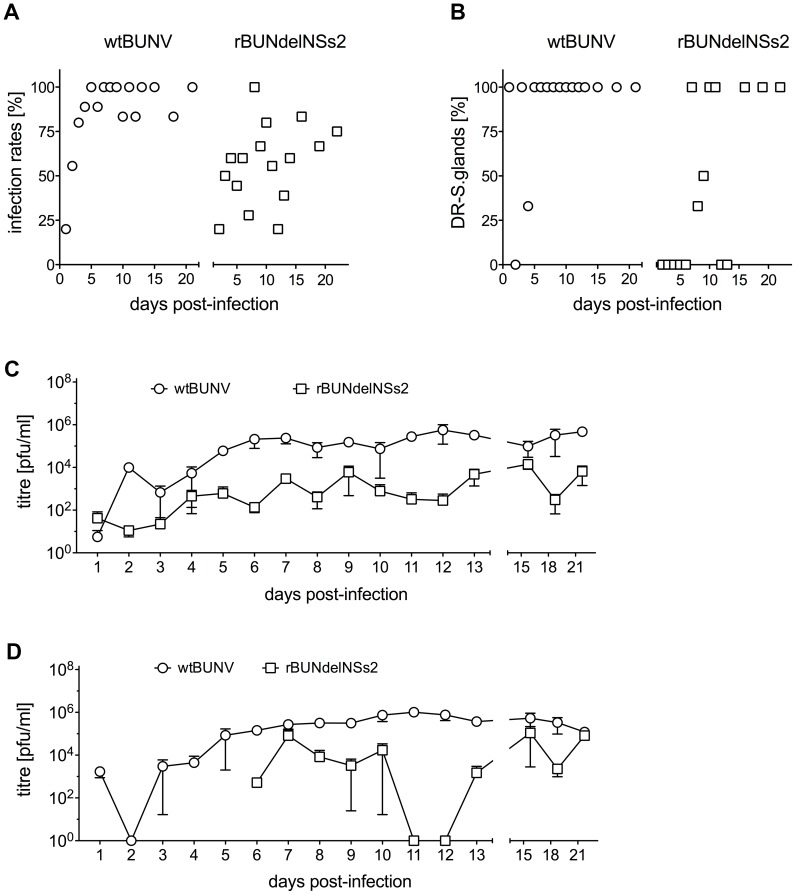
Virus replication in mosquito midguts and salivary glands. Following infection via blood-meal, 9 engorged mosquitoes were collected at days 1 to 13, and 6 engorged mosquitoes were collected at days 15, 18 and 21. Midguts and salivary glands were dissected, pooled into groups of 3 organs, and infectious virus determined by plaque assay. **A.** Midgut infection rates. The percentage of virus positive midguts over total number of tested mosquitoes was calculated. **B.** Average virus titre per mosquito midgut. Error bars show the standard error between the different pools. **C.** Disseminated infection rates. The percentage of virus positive salivary glands over total number of positive mosquito midguts was calculated. **D.** Average virus titres per mosquito salivary gland. Error bars show the standard error between the different pools.

To confirm that lack of NSs results in delayed BUNV replication, in another experiment, midguts and salivary glands were dissected from engorged mosquitoes and examined for presence of virus. At days 1 to 13 post-feeding, nine mosquitoes, and at days 15 to 21 post-feeding, six mosquitoes, were collected, and pools of three isolated organs were made. Infectious virus could be detected in the midgut by 2 days post-infection for both viruses. By 4 days post-feeding, midgut infection rates (calculated as percentage of virus positive midguts among engorged mosquitoes tested) for wtBUNV reached about 80%, and stayed at high levels for the duration of the experiment (Figure 8A). In comparison infection rates by rBUNdelNSs2 were significantly lower, and more variable. The titres of virus in midguts were also different between the viruses, with wtBUNV titres being about 100-fold higher than those of rBUNdelNSs2 (Figure 8B).

In order to be successfully transmitted by a mosquito vector to a vertebrate host, following replication in the midgut the virus must disseminate to secondary tissues like muscles, haemolymph, fat body and eventually the salivary glands. If the virus is found only in the midgut, infection is regarded as non-disseminated. Therefore the transmission potential of BUNV in infected mosquitoes was estimated by calculating disseminated infection rates to salivary glands. Both viruses managed to disseminate to salivary glands successfully, though with different kinetics. wtBUNV was detected in salivary glands by 3 days after feeding whereas the NSs-deletion mutant was not detected in salivary glands until 6 days post-feeding (Figure 7C). The titres of wtBUNV were generally higher in salivary glands than those of rBUNdelNSs2 (Figure 7D). These data suggest that the virus lacking NSs had more difficulty in overcoming the cellular defences in the midgut, but when rBUNdelNSs2 did manage to overcome the midgut escape barrier it was capable of spreading throughout the rest of the body, including to salivary glands.

## Discussion

The BUNV NSs protein has been widely studied in mammalian cells where it is has been shown to be a major virulence determinant. NSs counteracts the host innate immune response mainly by globally inhibiting RNA polymerase II-mediated transcription [16], [18]–[21]. On the other hand, BUNV NSs does not affect cellular transcription in infected mosquito cells [17], and for the related La Crosse orthobunyavirus (LACV), no specific function for its NSs protein was found in mosquito cell lines [23]. However, our results presented above show that the BUNV NSs protein could be a crucial factor for efficient infection in certain cultured mosquito cells and in live mosquitoes.

Comparison of wtBUNV and rBUNdelNSs2 showed that NSs is nonessential for replication and establishment of persistent infection in *Ae. albopictus* C6/36 and C7-10 cells. By contrast, rBUNdelNSs2 seemed unable to replicate productively in neither the *Ae. albopictus* U4.4 cell line nor the *Ae. aegypti* Ae cell line, indicating a requirement for the NSs protein. Unfortunately, attempts to express NSs exogenously in U4.4 cells, and thus enable rBUNdelNSs2 replication, have so far been unsuccessful. Comparison of the levels of released wtBUNV and the recombinant NSs deletion mutant suggests that NSs protein enables high-level virus replication in all cells except *Ae. albopictus* C7-10, where expression or not of NSs had little effect. It is becoming clearer that, much like with mammalian cell lines, different mosquito lines differ in their response to viral infection and their ability to reproduce accurately events in whole mosquitoes [31], [57], [58]. Our data suggest that of the *Ae. albopictus* lines, the U4.4 cell line could similarly be a good tissue culture model to study BUNV replication and the role of NSs in infection of mosquito cells.

The three phases of infection, early, acute and late, described by Lopez-Montero and Risco [27] in BUNV-infected C6/36 cells were also identified in the other two *Ae. albopictus* cell lines. These phases were characterized by changes in the location of viral proteins and changes of the cell morphology throughout each stage. Microscopic observations of the wild type and NSs-deleted viruses showed that lack of NSs reduced the extent to which mosquito cells underwent morphological changes during the acute stage of infection. Possibly these changes are driven by a defense mechanism that allows the cells to cope with severe viral infection, and Lopez-Montero and Risco [27] suggest that the filopodia-like projections could be involved in spreading “protective signals” among the cells. Branch-like projections have also been observed in cells infected with a rodent-transmitted bunyavirus, Sin Nombre hantavirus, and suggested to be sites where progeny particles were released [45], [59]. A release method that does not involve rupturing the cell membrane could explain why virus replication does not kill mosquito cells and persistence is maintained.

To date the only mosquito-borne bunyavirus that was shown to induce RNAi response in mosquito cells is La Crosse orthobunyavirus [23], [37], [39]. We have also detected, by conventional Northern blotting analysis, virus-specific small RNAs (<30 nucleotides) in all *Ae. albopictus* cell lines infected with wtBUNV (data not shown). Soldan et al. (2005) showed that La Crosse virus replication could be inhibited in C6/36 cells pre-treated with virus-specific small interfering RNAs [60]. Here we showed that an RNAi response could be efficient in inhibiting BUNV infection too. When we transfected cells with long virus-specific dsRNA, BUNV replication was only reduced in U4.4 cells, which have previously been shown to have fully functional Dicer 2 [37], [39], suggesting that the dsRNA was processed efficiently to generate small inhibitory RNAs. Bunyaviruses efficiently avoid dsRNA-based RNAi responses by coating their RNA segments with the nucleoprotein, thereby avoiding the formation of dsRNA species [61]. Our transfection experiments showed that if specific dsRNA species were produced in abundance, mosquito cells could overcome wtBUNV infection. Interestingly, the BUNV NSs deletion mutant was capable of efficient replication only in Dicer 2 incompetent cell lines. There are no studies showing involvement of any bunyavirus NSs protein in overcoming RNAi response in mosquito cells, but the NSs protein of La Crosse virus has been shown to inhibit RNAi antiviral activity in mammalian cells [60]. Further work is required to investigate whether BUNV NSs has an effect on mosquito Dicer 2 activity, or if exogenous expression of NSs in U4.4 cells would render them permissive for rBUNdelNSs2 replication.

The lack of genomic sequence data for the *Ae. albopictus* mosquito makes it a less attractive model in which to study host-virus interactions. Therefore, we investigated whether cells derived from *Ae. aegypti*, whose sequence has been determined [48], were permissive for BUNV replication. Our results showed that BUNV growth in Ae cells resembled that in U4.4 cells, and that the NSs protein also proved to be necessary for efficient replication. Thus *Ae. aegypti* cells could be a useful tool in studying BUNV infection and identification of cellular components that are important for viral replication.

There is only one study of BUNV replication in mosquitoes; Peers [52] reported that BUNV multiplied in the gut, disseminated to salivary glands and was transmitted to suckling mice in *Ae. aegypti* mosquitoes more efficiently than in *Ae. vexans* and *Ae. canadensis*. Our results for wtBUNV showed similar kinetics of viral replication and dissemination in *Ae. aegypti* to those obtained by Peers. Fewer mosquitoes were infected with the NSs-deletion mutant. In addition, we showed that the NSs protein contributes to high-level virus replication in that the mutant virus lacking NSs grew to lower titres. Similarly, the wild-type virus disseminated to salivary glands more efficiently than rBUNdelNSs2, and the lower levels of rBUNdelNSs2 in salivary glands could affect the transmission potential of the virus. This requires further investigation.

Our experiments showed that BUNV replication in *Ae. aegypti* mosquitoes resembled replication in *Ae. aegypti* Ae cells, as well as *Ae. albopictus* U4.4 cells. These data corroborate previous conclusions that these cell lines are the most appropriate mosquito cell culture models to study arbovirus infection. The NSs protein was required for efficient replication in both mosquito cells with a competent Dicer 2-RNAi system and in adult mosquitoes. In a proportion of mosquitoes, however, the mutant virus could eventually overcome host defences, though remained constrained as evidenced by lower virus titres. As BUNV is a relatively fast growing virus perhaps its reproduction rate is able to counteract the host's inhibitory responses. The NSs protein of Rift Valley fever phlebovirus (also in the family *Bunyaviridae*), although being quite distinct in size, amino acid sequence and expression strategy from BUNV NSs, plays a similar role in mammalian cells in overcoming innate immune responses via global shut-down of cellular transcription [62]. Two recent papers investigated the role of Rift Valley fever virus NSs in infection of mosquitoes, and neither observed any difference in infection or dissemination rates between wt and NSs-deleted viruses [63], [64]. Interestingly, deletion of another non-structural protein, NSm, from Rift Valley fever virus almost completely abolished its ability to replicate in mosquitoes [64]. These results illustrate the diversity and complexity of virus-host interactions within the *Bunyaviridae* family.
